# AGEs Decrease Insulin Synthesis in Pancreatic β-Cell by Repressing Pdx-1 Protein Expression at the Post-Translational Level

**DOI:** 10.1371/journal.pone.0018782

**Published:** 2011-04-18

**Authors:** Tingting Shu, Yunxia Zhu, Hongdong Wang, Yan Lin, Zhuo Ma, Xiao Han

**Affiliations:** 1 Key Laboratory of Human Functional Genomics of Jiangsu Province, Nanjing Medical University, Nanjing, Jiangsu, China; 2 Hubei University of Technology, Wuhan, Hubei, China; Pennington Biomedical Research Center, United States of America

## Abstract

Advanced glycation end products (AGEs) have been implicated in diverse pathological settings of many diabetic complications, and the possible mechanisms have been widely reported. However, the relationship between AGEs and pancreatic β-cell dysfunction is still poorly understood. Recent studies have shown that AGEs can impair β-cell function by inducing apoptosis or decreasing insulin secretion. Our previous research revealed that AGEs could significantly down-regulate insulin transcription and reduce β-cell glucose-stimulated insulin secretion (GSIS). Here, we investigated the possible mechanisms underlying AGE-related suppression of insulin synthesis. In the rat pancreatic β-cell line INS-1, we found that AGEs induced dephosphorylation of Foxo1 and increased its accumulation in the nucleus. The translocation of Foxo1 subsequently inhibited pancreatic-duodenal homeobox factor-1 (Pdx-1) levels in both nuclear and cytoplasmic compartments. We observed that with AGEs treatment, Pdx-1 protein levels decreased after 4 h, but there was no change in the Pdx-1 mRNA level or promoter activity at the same time point; this demonstrated that the decrease in Pdx-1 expression was not regulated at the transcriptional level. In our study, the decrease in Pdx-1 protein level was related to its reduced stability, overexpression of DN-Foxo1 could partially reverse the inhibition of Pdx-1 expression. Pretreatment with AGEs receptor (RAGE) antibody also prevented the AGE-induced diminution of Pdx-1 protein and insulin mRNA expression. In summary, AGEs induced nuclear accumulation of Foxo1; this in turn reduced Pdx-1 expression by decreasing its protein stability, ultimately affecting insulin synthesis.

## Introduction

Type 2 diabetes arises when the endocrine pancreas fail to secrete sufficient insulin to cope with metabolic demands, due to acquired β-cell secretory dysfunction and insulin synthesis suppression [1]. Glucotoxicity, lipotoxicity, and glucolipotoxicity are secondary phenomena that are proposed to play important roles in all forms of type 2 diabetes [2]. Advanced glycation end products (AGEs) in islets are an important mechanism for glucotoxicity [3]. A large body of evidence suggests that people with diabetes have higher levels of AGEs than nondiabetic subjects because of hyperglycemia [4], [5], [6]. Clinical investigations have indicated that AGEs are independently correlated with the proinsulin-to-insulin ratio (P/I ratio), which is a strong predictor of β-cell dysfunction [3]. In animal models, several diabetic complications, including diabetes-associated nephropathy [7], retinopathy [8], neuropathy [9], and atherosclerosis [10], have been linked to AGEs. Moreover, concrete mechanisms that link AGEs with these complications have been defined. However, little research has focused on the cytotoxic effects of AGEs on β-cell. Only a few studies have demonstrated that AGEs can impair β-cell secretory functions [11], lower insulin content [12], or induce apoptosis in INS-1 cells and primary islets [8], [13], [14]. A possible explanation for these observations is that AGEs might induce the expression of inducible nitric oxide synthase (iNOS), leading to inhibition of cytochrome *c* oxidase and ATP synthesis. Another possibility is the generation of reactive oxygen species (ROS) induced by AGEs. Currently, the relationship between AGEs and β-cell is still poorly defined; therefore, clearly establishing the role of AGEs in β-cell damage and further revealing the possible mechanisms responsible for this damage is very necessary.

Pdx-1 plays a significant role in both pancreatic development and maintenance of β-cell function [15]. Targeted disruption of this transcription factor in β-cell leads to diabetes, whereas reducing its expression affects insulin expression and secretion [16], [17]. Numerous studies have focused on Pdx-1, which is considered to be a principal insulin regulator, and the regulation mode between Pdx-1 and insulin has been clearly demonstrated. In Pdx-1 allele-inactivated (*Pdx-1^+/-^*) mice, β-cell develops hyperplasia but fails to increase insulin content [18]. Adenoviral expression of Pdx-1 in liver cells induced endogenous insulin expression, which had been confirmed to be biologically active [19]. In the INS-1 cell line and pancreatic islets, delivery of a small interfering RNA specific for Pdx-1 decreased insulin mRNA levels to approximately 40% of normal levels [20]. Current research suggests that a decrease in the expression of Pdx-1 will inevitably lead to a deficiency in insulin transcription, since the binding of Pdx-1 on the gene promoter of insulin will decline [21], [22].

Foxo1 is a transcription factor of the forkhead family that plays a critical role in cellular differentiation, proliferation, apoptosis, and stress resistance [23]. Foxo1 controls two important processes in the pathogenesis of type 2 diabetes: hepatic glucose production and β-cell compensation of insulin resistance [24]. Recent studies have revealed that Foxo1 negatively regulated Pdx-1 by modulating Pdx-1 subcellular localization or suppressing *Pdx-1* gene transcription [25]. The two transcription factors exhibit mutually exclusive patterns of nuclear localization in β-cell; constitutive nuclear expression of a mutant Foxo1 is associated with the lack of Pdx-1 expression [26], [27]. However, the type of molecular events that occur between Foxo1 and Pdx-1 remains unclear. Here, we propose that Foxo1 may contribute to the decrease in Pdx-1 stability under AGEs stimulation; this may provide a new regulation model for the interaction between these factors.

In this study, we utilize the insulin-secreting β-cell line INS-1 to test the hypothesis that AGEs may down-regulate Pdx-1 protein stability; this in turn leads to a reduction in Pdx-1 protein levels, ultimately contributing to a deficiency in insulin synthesis.

## Results

### The effects of GS on insulin promoter activity, transcription, and content

**Figure 1:**
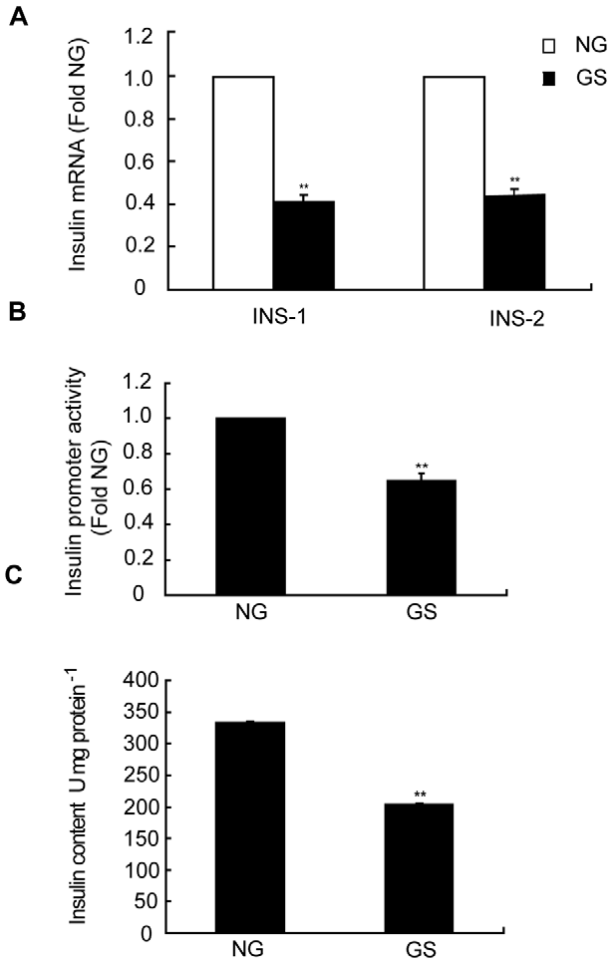
Glycated serum (GS) inhibited insulin synthesis. (A) Real-time PCR analysis was performed to measure Insulin1 and Insulin2 mRNA 24 h after treatment with 10% GS. Relative quantification was used to calculate the change in insulin mRNA, which was depicted as the fold change. (B) INS-1 cells transfected with pGL3-insulin constructs for 24 h, followed by treatment with nonglycated (NG) or 10% GS for a further 24 h. Lysates were harvested for the luciferase assay. (C) INS-1 cells cultured with NG or 10% GS for 24 h. Insulin content was measured after acidified ethanol extraction. Data are represented of three separate experiments. **p<0.01 vs. NG.

The transcription level and content of insulin are important indices used to evaluate the function of pancreatic β-cell. Inhibition of insulin transcription leads to a deficiency in its synthesis, and thus, the glucose metabolic balance cannot be maintained. In the present study, when we treated the INS-1 cells with GS for 24 h, Insulin1 and Insulin2 transcription was greatly repressed. As shown by the real-time PCR results, Insulin1 and 2 mRNA expression sharply decreased to 32% and 38%, respectively (Fig. 1A). In addition, we examined the effects of GS on insulin promoter activity by using the luciferase reporter gene assay. Compared to the NG-treated cells, the GS-treated cells showed an approximately 35% decrease in normalized insulin promoter luciferase activity (Fig. 1B). The total cellular insulin content was also significantly decreased in the GS group (Fig. 1C).

### GS inhibited Pdx-1 protein expression

**Figure 2:**
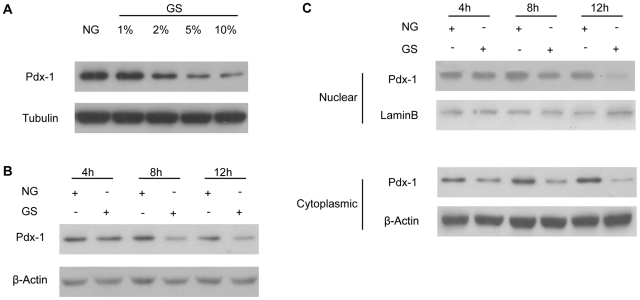
Glycated serum (GS) inhibited Pdx-1 protein expression. (A) INS-1 cells were treated with nonglycated (NG; control) serum or with varying concentrations of GS for 24 h and then harvested for western blot analysis. (B) INS-1 cells were treated with NG or 10% GS and harvested at the indicated time point for western blot analysis. (C) INS-1 cells were treated with NG or 10% GS for 4, 8, or 12 h. Cytoplasmic and nuclear protein was extracted, and then Pdx-1 determination was conducted.

Although many transcription factors have been implicated in the regulation of insulin transcription, three β-cell-specific transcription regulators, namely, Pdx-1, neurogenic differentiation 1 (NeuroD1), and V-maf musculoaponeurotic fibrosarcoma oncogene homologue A (MafA), have been demonstrated to play a crucial role in insulin transcription regulation and pancreatic β-cell function. We determined the effects of GS on all of these transcription factors and found that Pdx-1 expression was most inhibited. As shown in (Fig. 2A), Pdx-1 protein levels decreased in a dose-dependent manner. The levels of Pdx-1 protein in INS-1 cells treated with GS also appeared to decrease in a time-dependent manner (Fig. 2B). Since Pdx-1, as a transcription factor, can shuttle between the nucleus and cytoplasm and its activity is directly related to its level in the nucleus, we next examined the intracellular localization of Pdx-1 after GS stimulation. Consistent with our finding of a reduction in total Pdx-1 protein expression, Pdx-1 protein levels decreased both in nuclear and cytoplasmic extracts at 4 h, and persisted to 12 h (Fig. 2C).

### GS down-regulated Pdx-1 at the post-translational level

**Figure 3:**
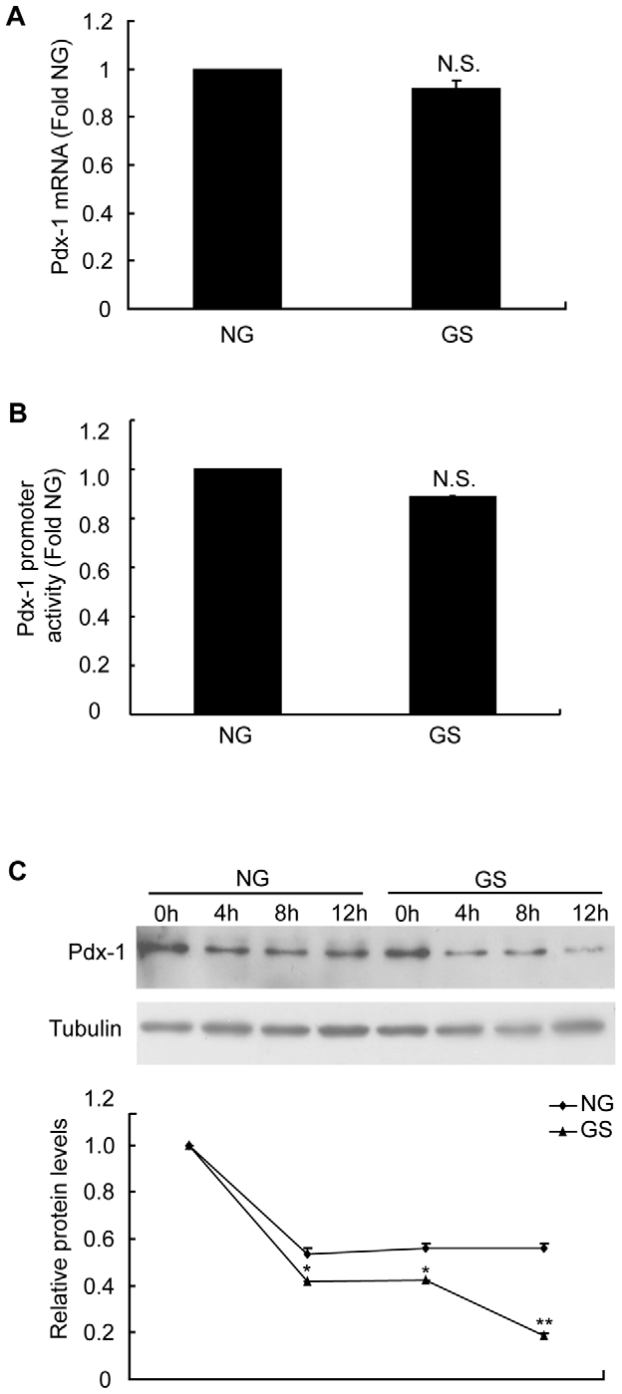
Glycated serum (GS) down-regulated Pdx-1 at the post-translational level. (A) Real-time PCR analysis was performed to measure Pdx-1 mRNA 4 h after treatment with nonglycated (NG) or 10% GS. (B) INS-1 cells were transiently transfected with the pGL3-Pdx-1 construct for 24 h. Luciferase activity was assayed after a 24 h incubation with NG or 10% GS. (C) INS-1 cells were treated with NG or 10% GS together with cyclocheximide (50 μg/mL) for the indicated periods of time. All the treated cells were then harvested and lysed for western blot analyses. Representative immunoblots and a graph showing protein levels of Pdx-1 relative to α-tubulin are presented. *p<0.05 vs. NG; **p<0.01 vs. NG.

To further explore the mechanism of GS-induced reduction of Pdx-1 expression, we determined the effects of GS on Pdx-1 mRNA expression and promoter activity. With GS treatment for 4 h, Pdx-1 mRNA levels showed no difference compared with NG group (Fig. 3A), but Pdx-1 protein levels were reduced at this time point (Fig. 2B). We transfected INS-1 cells with the pGL3-Pdx-1 luciferase reporter construct, then treated them with NG or GS and found that GS had no influence on Pdx-1 promoter activity either (Fig. 3B). On the basis of these results, we suspected that GS would negatively affect Pdx-1 protein stability. To confirm this possibility, we evaluated Pdx-1 protein expression stability in cells treated with NG or GS for 0, 4, 8, and 12 h; protein synthesis was inhibited by using cycloheximide. After de novo protein synthesis was blocked, Pdx-1 protein levels decreased more rapidly with GS stimulation. These results suggested that GS regulated Pdx-1 expression at the post-translational level by increasing its degradation.

### GS treatment resulted in Foxo1 nuclear accumulation contributing to a decrease in Pdx-1 stability

**Figure 4:**
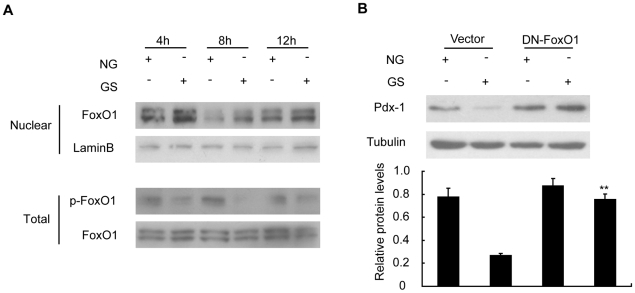
Glycated serum (GS) promoted Foxo1 nuclear accumulation to reduce Pdx-1 stability. (A) INS-1 cells were treated with nonglycated (NG) or 10% GS for the indicated periods of time. All the cells were collected for total protein and nuclear protein extraction. Western blot assay was used to detect the phosphorylated-Foxo1, total Foxo1, and nuclear Foxo1. (B) INS-1 cells overexpressing DN-Foxo1 construct were cultured for 24 h and then treated with nonglycated (NG) or 10% glycated serum (GS) for another 24 h. Pdx-1 protein level was determined by western blot assay. Representative immunoblots and a graph showing the protein levels of Pdx-1 relative to α-tubulin are presented. Data are represented of three separate experiments. **p<0.01 vs. GS, vector-transfected group.

Pdx-1 is potentially modified by several post-translational mechanisms such as phosphorylation, glycosylation, and sumoylation. However, the exact underlying mechanism remains largely unknown. Considering direct regulation and excluding co-localization between Foxo1 and Pdx-1, there is a high likelihood that AGEs decrease Pdx-1 protein stability via Foxo1. First, we aimed to confirm that Foxo1 was indeed activated by GS in INS-1 cells. We treated INS-1 cells with NG and GS for 4, 8, and 12 h. The cells were then collected for protein extraction and western blot analyses. The phosphorylation levels of Foxo1 reduced in a time-dependent manner, whereas the nuclear accumulation of Foxo1 increased (Fig. 4A). To ensure the involvement of Foxo1 in the process of GS-induced decline in Pdx-1 expression, we overexpressed DN-Foxo1, which could compete with endogenous Foxo1. After a 24 h transfection, the transfected cells were treated with NG or GS for a further 24 h. In this case, the reduction in Pdx-1 protein expression was greatly reversed (Fig. 4B). These data suggested that the decrease in Pdx-1 protein stability caused by GS exposure was due to the nuclear accumulation of Foxo1.

### Inhibition of RAGE blocked the effects of GS on Pdx-1 expression and insulin transcription

**Figure 5:**
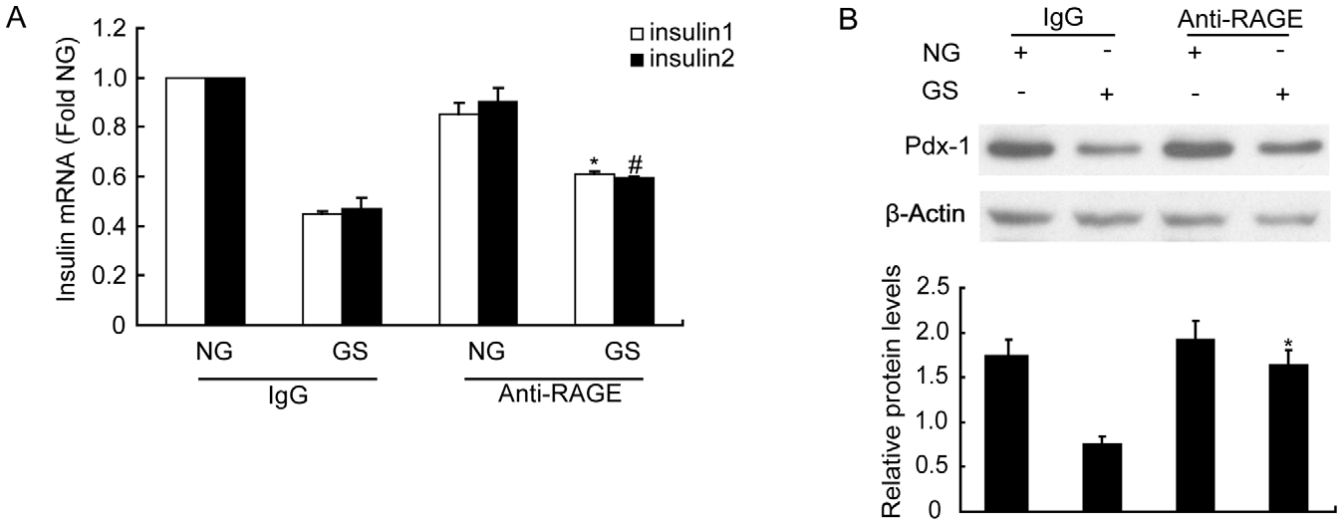
RAGE antibody reversed glycated serum (GS)-induced impairment of Pdx-1 protein expression and insulin mRNA expression. (A) INS-1 cells were pretreated with RAGE antibody for 1 h and then treated with nonglycated (NG) or 10% GS for a further 24 h. Insulin mRNA was measured by real-time PCR assay. (B) With the same treatment, Pdx-1 protein level was determined by western blot assay. Representative immunoblots and a graph showing the protein levels of Pdx-1 relative to β-actin are presented. Data are represented of three separate experiments. *p<0.05 vs. the GS IgG Insulin1 group; #p<0.05 vs. the GS IgG Insulin2 group; *p<0.05 vs. the GS IgG group.

RAGE can mediate AGEs signaling in many cell types; however, its role in pancreatic β-cell, particularly in insulin synthesis deficiency caused by GS stimulation in INS-1 cells, is not clear. We therefore pretreated cells with RAGE antibody for 1 h to block RAGE activity, and then treated the cells with NG or GS for a further 24 h. As shown by the results of real-time PCR (Fig. 5A), RAGE antibody partially reversed the decrease in insulin mRNA levels under GS treatment; however, the antibody had no effect on the expression of insulin. Furthermore, in the presence of RAGE antibody, the decrease in Pdx-1 protein was almost completely reversed. No protective effect on Pdx-1 expression was observed when cells were co-incubated with IgG (Fig. 5B).

## Discussion

In the present study, we demonstrated that GS treatment could cause a sharp decrease in Pdx-1 protein levels. Since there was no change in Pdx-1 mRNA and promoter activity, but there was an increase in the protein degradation rate. Therefore, we suggested that GS down-regulated Pdx-1 protein expression at a post-translational level. The decrease in Pdx-1 stability was related to the accumulation of Foxo1 in the nucleus and could be reversed by overexpression of DN-Foxo1. Ultimately, Pdx-1 expression deficiency would lead to a decrease in insulin mRNA, promoter activity and content.

We and other researchers have previously shown that AGEs can damage pancreatic β-cell function by decreasing cell viability, thereby reducing insulin secretion and content. Zhao *et al.* have reported that AGEs inhibited cytochrome *c* oxidase and ATP production; this could be a possible explanation for the defect in insulin secretion. Minsu *et al.* have attributed this loss of β-cell function to an increased production of ROS. However, little is known about the connection between AGEs and the obstacle to insulin synthesis. Here, we showed that GS can significantly down-regulated Pdx-1 protein levels, which in turn led to the inhibition of its role in promoting insulin transcription.

Eukaryotic cell protein synthesis is under complex regulation. Gene transcription constitutes only a part of the regulatory mechanism. Therefore, other control mechanisms, in particular post-translational modification of proteins, may be particularly important. Pdx-1 plays a crucial role in β-cell function, and a number of studies have indicated that it could be regulated at the transcriptional, post-transcriptional, and post-translational level. Robertson *et al.* have shown that the post-transcriptional loss of Pdx-1 mRNA is the primary mechanism for the loss of Pdx-1 protein induced by oxidative stress in HIT-T15 cells [28]. Two serine residues (Ser^61^ and Ser^66^) are located in the transactivation domain of Pdx-1 and are conserved among different species [17]. We observed that there was no decrease in Pdx-1 mRNA level and promoter activity compared with NG group, but at the same time, Pdx-1 protein expression was strongly repressed. Furthermore, as de novo protein synthesis was inhibited by cyclocheximide, GS could induce more rapid degradation of Pdx-1; this suggested that GS clearly down-regulated Pdx-1 expression at the post-translational level. However, the exact post-translational modification involved in, the conditions when this occurs, the underlying mechanism, and the effect on Pdx-1 function remain largely unresolved. Previous studies have shown that phosphorylation of Ser^61^ and Ser^66^ could increase the rate of Pdx-1 degradation and decrease the half-life of Pdx-1 protein. In our investigation, the question of whether the reduced stability of Pdx-1 is related to an increased phosphorylation of these two sites needs to be further explored.

We revealed that the diminished level of Pdx-1 was associated with Foxo1 nuclear accumulation. With GS treatment, Foxo1 dephosphorylation increased, and it was shuttled to the nucleus. The mechanism of GS-induced Foxo1 nuclear accumulation is completely unknown. Kawamori *et al.* have reported that under oxidative stress conditions, activation of the c-Jun N-terminal kinase (JNK) pathway induced the nuclear and cytoplasmic transposition of Pdx-1. Foxo1 played a role as a mediator between the JNK pathway and Pdx-1. However, GS could not activate the JNK pathway in our experiment. Only a minor increase in ROS was observed, and this suggested that the decreased expression of Pdx-1 was probably not related to oxidative stress. Indeed, oxidative stress is thought to be the main causal factor in the pathogenesis of diabetic complications related to AGEs. The possible reason for such weak oxidative stress in our study could be the preparation of GS. Compared with glucose, ribose is probably a much stronger oxidant and can cause oxidative stress in the islets very quickly, whereas many months of culture may be required to induce oxidative stress with high glucose [3].

RAGE antibody partially reversed the defective of Pdx-1 expression and insulin biosynthesis. According to this partially protective effects, it is important to understand the relationship between AGEs and their receptors. AGE-R1 is largely responsible for the clearing of AGEs. The role of AGE-R2, R3, and the scavenger receptors (SR-B CD36) are less well defined [4], [29]. RAGE and other receptors appear to mediate cell signaling and activate a stress response leading to cellular dysfunction [30]. RAGE, as a member of the immunoglobulin superfamily, is expressed in a few cell types only. We have confirmed that the *RAGE* gene is present in rat pancreatic β-cell (INS-1 cells) and its expression increased with GS stimulation. Pretreatment of cells with RAGE antibody for 1 h partially prevented the decrease in insulin mRNA and Pdx-1 protein expression. The reason why RAGE antibody does not fully block Pdx-1 and insulin decrease is probably due to the complexity of the AGE receptor system. Many receptors involve in and these receptors occupy both positive and negative roles in actions of AGEs. Therefore, it is difficult to determine which receptors participate in the process of AGE-related deterioration of insulin synthesis. Although RAGE plays a prominent role in this pathway, it is not the only receptor that is involved. The cytosolic domain of RAGE is critical for RAGE-dependent signal transduction; however, only Dia-1 and ERK have been confirmed to interact with RAGE [31], [32]. In our study, the gap between RAGE and Foxo1 still needs to be further researched.

In conclusion, we demonstrate that in addition to causing diabetic complications, AGEs can directly impair pancreatic β-cell function by inhibiting insulin synthesis. We attribute these effects to a reduction in the expression of the key insulin transcription factor Pdx-1. Pdx-1 protein expression deficiency is not due to the inhibition of its transcription, but due to the decrease in its protein stability. Thus, the present study uncovers a new mechanism that might contribute to the AGE-induced inhibition of insulin synthesis.

## Materials and Methods

### AGE-fetal bovine serum preparation

Glycated serum (GS) was prepared as described previously [30]. Fetal bovine serum (FBS; Hyclone, Logan, UT) was incubated under sterile conditions with d-glucose (90 g/L) at 37°C for 3 weeks. Unincorporated sugars were then removed by dialysis against phosphate-buffered saline (PBS). Control nonglycated serum (NG) was incubated under the same conditions but without d-glucose. Application of the *Limulus* amebocyte assay before the *in vitro* study revealed that the reagents contained less than 0.2 ng/mL of endotoxin.

### Cell culture

INS-1 cells, a rat insulinoma cell line [14], [33], were cultured in RPMI-1640 medium (Invitrogen, Grand Island, NY) with 11.1 mM d-glucose supplemented with 10% FBS, 100 U/mL penicillin, 100 µg/mL streptomycin, 10 mM HEPES, 2 mM l-glutamine, 1 mM sodium pyruvate, and 50 µM β-mercaptoethanol (Sigma-Aldrich, St. Louis, MO). All cell culturing was performed in a Thermo tissue-culture incubator that provided an environment of 95% O_2_/5% CO_2_ gas. Before addition of GS, the cells were gently washed in PBS. NG or GS was added to the appropriate experiments. Cells were incubated for an additional indicated time.

### Real-time RT-PCR

INS-1 cells were cultured and treated as described above. Total RNA was extracted using Trizol reagent (Invitrogen). First-strand cDNA synthesis was performed using 1 μg of total RNA and an avian myeloblastosis virus reverse transcription system. The primers were designed using the software Primer Express (Applied Biosystems, Foster City, CA). Real-time quantitative PCR was performed using the SYBR Green PCR Master Mix and ABI Prism 7000 Sequence Detection System (Applied Biosystems). All data was analyzed using the expression of β-actin as a reference.

### Plasmid construction

The pGL3-insulin, pGL3-Pdx-1, and DN-Foxo1 plasmids described in this study were available in our laboratory. The two luciferase reporter constructs contain -2000 bp of insulin promoter or homology region 2 (*PH2*) domain of the Pdx-1 promoter [34]. The DN-Foxo1 construct lacks a transactivation domain; therefore, although it cannot activate its target, it can bind its target gene [25].

### Western blot analysis

INS-1 cells were cultured and treated as described above, then lysed with ice-cold lysis buffer containing the following reagents: 50 mM Tris-HCl (pH 7.4), 1% NP-40, 150 mM NaCl, 1 mM EDTA, 1 mM phenylmethylsulfonyl fluoride, and complete proteinase inhibitor (one tablet per 10 mL; Roche Molecular Biochemicals). After protein content determination, western blotting was performed as described previously [35]. Individual immunoblots were probed with antibodies to mouse anti-RAGE monoclonal antibody (Santa Cruz Biotechnology) diluted to 1∶800, rabbit anti-Pdx-1 (Upstate) monoclonal antibody diluted to 1∶4000, rabbit anti-phosphorylation-Foxo1 (Cell Signalling) monoclonal antibody diluted to 1∶800, or rabbit anti-Foxo1 (Santa Cruz Biotechnology) monoclonal antibody diluted to 1∶1000. Target protein levels were quantified relative to the levels of the control protein, mouse anti-β-actin (Sigma-Aldrich, St. Louis, MO) monoclonal antibody diluted to 1∶5000, mouse anti-α-tubulin (Sigma-Aldrich) monoclonal antibody diluted to 1∶5000, and goat anti-lamin B (Santa Cruz Biotechnology) monoclonal antibody diluted to 1∶800.

### Insulin content

INS-1 cells were seeded in 24-well plates. After 24 h, cells were washed twice with PBS (pH 7.4) at 0°C and extracted with acid/ethanol (0.15 M HCl in 75% ethanol in H_2_O) for 16 h at 0°C. Supernatants were collected and stored at −80°C until insulin determination was carried out by ELISA. The results were normalized to the total protein concentration.

### Statistical analysis

Comparisons were performed using Student's *t-*test between pairs of groups, or ANOVA for multiple group comparison. Results are presented as means ± SEM. A *p* value of less than 0.05 was considered to be statistically significant.
